# Involvement of CD95 and ligand in CD4+ T-cell and CD8+ T-cell depletion and hepatic cytolysis in patients with chronic viral hepatitis B

**DOI:** 10.4102/ajlm.v10i1.1224

**Published:** 2021-03-15

**Authors:** Franklin S. Azebaze Agueguia, Paul Talla, Marie C. Okomo Assoumou, Graeme B. Jacobs, Cedric H. Mbakam, Elise Guiedem, Martha Tongo Mesembe, Emilia Lyonga, George Mondinde Ikomey

**Affiliations:** 1 Center for the Study and Control of Communicable Diseases (CSCCD), Faculty of Medicine and Biological Sciences (FMBS), University of Yaoundé 1 (UY1), Yaoundé, Cameroon; 2 Department of Internal Medicine and Specialties, Faculty of Medicine and Biomedical Sciences (FMBS), Yaoundé General Hospital, University of Yaoundé 1 (UY1), Yaoundé, Cameroon; 3 Department of Microbiology, Haematology, Parasitology and Infectious Diseases, Faculty of Medicine and Biomedical Sciences (FMBS), University of Yaoundé 1 (UY1), Yaoundé, Cameroon; 4 Division of Medical Virology, Department of Pathology, Faculty of Medicine and Health Sciences, Stellenbosch University, Tygerberg, South Africa

**Keywords:** HBV, CD95-CD95L, CD4+ T-cells, CD8+ T-cells, cirrhosis

## Abstract

**Background:**

Chronic viral hepatitis B (HBV) is characterised by progressive hepatocyte destruction and T-cell depletion. The mechanisms of the CD95-CD95 ligand (CD95L) signalling pathway during this chronic disease and the cirrhotic process remains unclear.

**Objective:**

We evaluated the involvement of the CD95-CD95L receptor-ligand system in T-cell depletion and hepatic cytolysis in patients with chronic HBV.

**Methods:**

This was a cross-sectional study conducted from September to December 2018 at the Yaoundé General Hospital, Cameroon. Four mL of whole blood was collected and analysed. The CD95 and CD95L levels, as well as the CD4+ T-cell and CD8+ T-cell counts, were performed by enzyme-linked immunosorbent assay and flow cytometry.

**Results:**

Of the 130 HBV-positive patients, 36 (27.7%) were cirrhotic and 94 (72.3%) were non-cirrhotic. The cirrhotic patients had significantly elevated CD95 (*p* < 0.001) and CD95L (*p* = 0.001) plasma levels, compared with non-cirrhotic patients. The CD4/CD8 ratios were lower in cirrhotic patients, compared to non-cirrhotic patients (*p* < 0.001). There were statistically significant correlations between CD95 level and CD4^+^ T-cell counts, between CD95 level and CD8^+^ T-cell counts, between CD95 level and the CD4/CD8 ratio, between CD95 level and fibrosis score, and between CD95L level and fibrosis score.

**Conclusion:**

CD95 and CD95L could be involved in T-cell depletion and hepatic cytolysis during the pathogenesis of chronic HBV and could potentially be used as biomarkers for immunological and hepatic monitoring in patients with chronic HBV.

## Introduction

The hepatitis B virus (HBV) is the leading cause of chronic liver disease and remains a major global health burden.^1^ Chronic HBV infection is denoted by the persistence of the HBV surface antigen in the host for at least 6 months.^1^ Viral hepatitis B is characterised by the progressive onset of fibrotic hepatic lesions; the pathogenesis remains unclear.^2^ Counts of CD4^+^ and CD8^+^ T-cells, which play an indispensable role in the resolution of HBV infection,^2^ are significantly lower in chronic HBV, but the mechanisms resulting in their depletion remain unclear.^3^

The severity of liver lesions caused by infection varies according to the immune status of the patient.^2^ An appropriate immune response leads to the necrosis of infected hepatocytes and the elimination of the virus.^1^ An excessive immune response induces massive hepatocyte destruction, while immunotolerance is marked by abundant but asymptomatic viral multiplication without destruction of hepatocytes.^3^ In chronic carriers of HBV, this immune response exists, but it is insufficient to eliminate the virus.^3^

The persistence of HBV in hepatocytes leads to repeated attempts to eliminate them by T-cells.^2^ This is mediated by several immune mechanisms, including apoptosis, which is a physiological process of cell death, implementing an actual cell lysis programme.^3^ The triggering of this ‘programmed death’ is done through the activation of specialised signalling pathways, namely the CD95-CD95 ligand (CD95L) pathway.^3^

CD95 (Fas or APO-1) is the receptor of CD95L (Fas ligand, FasL); CD95 belongs to the tumor necrosis factor-receptor/nerve growth factor-receptor (TNF-R/NGF-R) family.^3^ Members of this family are characterised by the presence of cysteine-rich domains in their extracytoplasmic portion; CD95 has three cysteine-rich domains.^3^ The ‘death receptor’ subfamily is distinguished at the intracytoplasmic portion that contains a domain of about 80 amino acids called the ‘death domain’.^3^

The induction of apoptosis by CD95 follows its oligomerisation by an agonist monoclonal antibody or by its natural ligand, CD95L.^3^ The latter belongs to the superfamily of TNF/NGF.^3^ The CD95 molecule is expressed in many tissues including the liver, the heart and the hematopoietic tissues.^3^ However, a total absence of expression of the CD95 protein in humans has no direct repercussions on the lymphoid system.^4^ CD95 is expressed on the surface membrane of activated T-lymphocytes or B-lymphocytes, during the first activation.^5^ After a second activation, the T-lymphocyte expresses CD95L and kills the activated lymphocytes (death in ‘trans’), including itself (death in ‘cis’), which is called ‘activation-induced cell death’ (AICD).^5^ The CD95-CD95L interaction, therefore, represents a route of control of the immune response, and the regulation of autoimmune proliferations.^5^ CD95-mediated T-cell apoptosis is a well-known mechanism for the prevention of immunopathogenesis and the maintenance of immune tolerance through the contraction of T-cell responses. This is described as an immune checkpoint mechanism.^5^ Three mechanisms maintain peripheral T-cells homeostasis: T-cells anergy, suppression by regulatory T-cells and AICD.^6^ The population of effector T-cells is controlled by AICD, which is initiated by repeated stimulation of the T-cell receptor via apoptosis mediated by CD95.^7^

Thus, AICD mediated by CD95 plays a very important role within the peripheral immune system.^6^ The mechanisms of the CD95-CD95L apoptotic signalling pathway during chronic HBV remains to be elucidated.^6^ Our objective was to evaluate the involvement of CD95-CD95L receptor-ligands in T-cell depletion and hepatic cytolysis in patients with chronic HBV.

## Methods

### Ethical considerations

Ethical approval to conduct the study was obtained from the Institutional Ethics and Research Committee for Human Health, of the Catholic University of Central Africa (No. 2019/0803/CEIRSH/ESS/MIM). Written and verbal informed consent was given by all participants. The study was conducted according to the ethical principles and guidelines of the international Declaration of Helsinki 2013. All procedures were standard and presented a minimal risk to participants. The names of the participants were replaced by alphanumeric codes and kept in a safe place, accessible only by the principal investigator. The physical documents were kept in secure locked drawers within the processing site. The digital documents were encrypted and secured by passwords and stored on secure hard disks.

### Study design

We performed a cross-sectional study from September to December 2018 of patients with chronic HBV infection. Participants received consultation at the Hepato-gastroenterology Department of the Yaoundé General Hospital, Cameroon. Patient recruitment was consecutive and non-probabilistic. The results of the biological analysis were returned to the patients and incorporated into their medical records.

### Study population

From the fibro test examination, patients were divided based on their METAVIR fibrosis score into cirrhotic and non-cirrhotic HBV-infected patients. Recruited patients were between 18 and 60 years of age. Patients with liver diseases of other aetiologies or those having HBV infection and a history of autoimmunity, drug-dependence or co-infection with other viruses, including hepatitis C virus (HCV), hepatitis D virus (HDV), HIV and human T-cell leukemia virus type 1, were excluded from the study.

The selection criteria for the control participants were: not having a medical history of HBV, HCV, HDV, human T-cell leukemia virus type 1 or HIV infection, autoimmunity and not having consumed alcohol in the past 7 years. These control participants were selected from a population of blood donors at the Yaoundé General Hospital and screened for these viral infections and diseases.

Social characteristics (age and sex) and clinical information (cirrhosis statute, levels of CD95 and CD95L, counts of CD4^+^ T-cells, CD8^+^ T-cells and CD4/CD8 ratio) were collected for each participant using a standard questionnaire.

### Sample collection and analysis site

Four millilitres of whole blood was collected into ethylene diamine tetra acetate acid anticoagulant tubes (Greiner Bio-One International GmbH, Kremsmünster, Austria), and transported at room temperature to the Immuno-virology Laboratory of the Center for the Study and Control of Communicable Diseases of the Faculty of Medicine and Biomedical Sciences of the University of Yaoundé 1. These samples were analysed within 24 h for CD4^+^ T-cells, CD8^+^ T-cells and the CD4/CD8 ratio. Plasma was obtained by centrifugation of whole blood at a speed of 5000 revolutions per minute for 5 min and aliquoted in cryovials. Plasma samples were frozen at −20 °C, for the subsequent determination of CD95 and CD95L levels. METAVIR scores of fibrosis were collected from patients’ medical records.

### Determination of CD4^+^ T-cell and CD8^+^ T-cell count and the CD4/CD8 ratio

We used the whole blood collected for the determination of CD4^+^ T-cells, CD8^+^ T-cells and the CD4/CD8 ratio. This was performed by flow cytometry, which is a technology that provides rapid multi-parametric analysis of single cells in solution, with specific fluorochrome-conjugated antibodies, using the Becton Dickenson fluorescent activated cells sorting system (Biosource, San Jose, California, United States). Results were obtained automatically for the absolute CD4^+^ T-cells and CD8^+^ T-cells count, including the CD4/CD8 ratio. This was done according to the manufacturer’s recommendations.

### Determination of CD95 and CD95L plasma level

The determination of the CD95 and CD95L plasma levels were performed by the sandwich enzyme-linked immunosorbent assay (ELISA) technique (Quantikine^®^, R&D Systems, Abingdon, United Kingdom), following the manufacturer’s instruction. Optical densities were measured at 450 nm using an ELISA reader (Sunrise Tecan, Tecan GmbH, Grödig/Salzburg, Austria), and all samples were assayed in duplicate. The CD95 and CD95L levels in the samples were determined by extrapolating the results from a standard curve.

### Statistical analysis

Data from this study were recorded in Microsoft Office Excel 2016, and statistical analysis was performed using EpiInfo^®^ 7.0 (Division of Public Health Surveillance and Informatics Epidemiology Program Office, MS K74 Centers for Disease Control and Prevention, Atlanta, Georgia, United States) and Graph Pad PRISM 5.0 (Graph Pad Software Inc., La Jolla, California, United States). Comparisons between CD4^+^ T-cells, CD8^+^ T-cells, CD4/CD8 ratio, CD95 and CD95L between different groups were performed using the non-parametric test of Mann-Whitney and Kruskal-Wallis. The correlations between CD95, CD95L, CD4^+^ T-cells, CD8^+^ T-cells, CD4/CD8 ratio and fibrosis score were established using the Spearman’s correlation coefficient (*r*). All values of *p* under 0.05 were considered statistically significant at a confidence interval of 95%.

## Results

For 190 patients recruited, 130 (68.42%) were HBV-infected patients, and 60 (31.58%) were control participants. Among the control participants, 36 (60.00%) were male and 24 (40.00%) were female, with an average age of 35 ± 13 years. Of the 130 HBV-infected patients, 36 (27.70%) were cirrhotic and 94 (72.30%) were non-cirrhotic. Among the cirrhotic patients, 23 (64.00%) were male and 13 (36.00%) were female, with an average age of 38 ± 16 years. Among non-cirrhotic patients, 52 (55.3%) were male and 42 (44.70%) were female, with an average age of 31.54 ± 9 years (Table 1).

**TABLE 1:** Social and clinical characteristics of hepatitis B virus-positive patients at Yaoundé General Hospital, Cameroon, September–December 2018.

Study population	*n*	%	Mean ± SD	Clinical status					
				Cirrhotic			Non-cirrhotic		
				*n*	%	Mean ± SD	*n*	%	Mean ± SD
**HBV-positive patients (*N* = 130)**	-	-	-	36	27.7	-	94	72.3	-
**Sex**	-	-	-	-	-	-	-	-	-
Male	75	57.7	-	23	64.0	-	52	55.3	-
Female	55	42.3	-	13	36.0	-	42	44.7	-
**Age (years)**	-	-	-	-	-	38.03 ± 16.11	-	-	31.54 ± 9.27
**Control participants (*N* = 60)**	-	-	-	-	-	-	-	-	-
**Sex**	-	-	-	-	-	-	-	-	-
Male	36	60	-	-	-	-	-	-	-
Female	24	40	-	-	-	-	-	-	-
**Age (years)**	-	-	35 ± 13	-	-	-	-	-	-

HBV, hepatitis B virus; SD, standard deviation.

**FIGURE 1:**
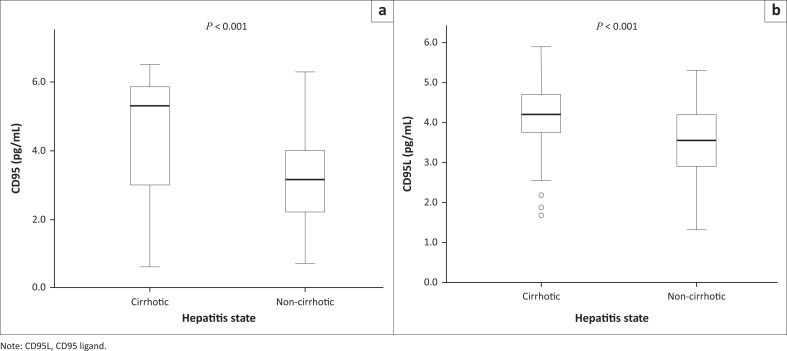
Level of CD95 and CD95L in cirrhotic and non-cirrhotic hepatitis B virus-positive patients at Yaoundé General Hospital, Cameroon, September–December 2018.

The level of CD95 in cirrhotic patients ranged from 0.6 picograms per millilitre (pg/mL) to 6.5 pg/mL, with a median of 5.3 pg/mL. The level of CD95 in non-cirrhotic patients ranged from 0.7 pg/mL to 6.3 pg/mL, with a median of 3.2 pg/mL. The difference between the CD95 levels of the cirrhotic and non-cirrhotic patients was statistically significant, with a *p*-value of less than 0.001 (Figure 1a). The level of CD95L in cirrhotic patients ranged from 1.8 pg/mL to 5.9 pg/mL, with a median of 4.2 pg/mL. The level of CD95L in non-cirrhotic patients ranged from 1.3 pg/mL to 5.3 pg/mL, with a median of 3.6 pg/mL. The difference between the CD95L levels of the cirrhotic and non-cirrhotic patients was statistically significant, with a *p*-value of 0.001 (Figure 1b).

**FIGURE 2:**
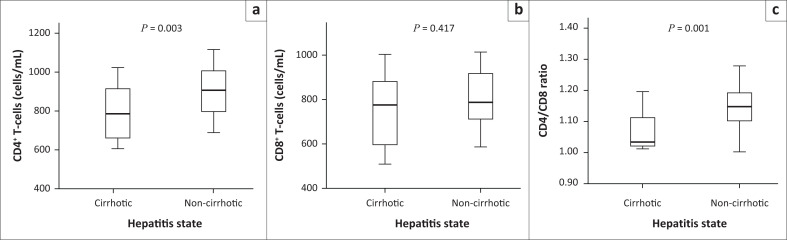
Value of CD4^+^ T-cells, CD8^+^ T-cells and the CD4/CD8 ratio in cirrhotic and non-cirrhotic hepatitis B virus-positive patients at Yaoundé General Hospital, Cameroon, September–December 2018.

The CD4^+^ T-cell values in cirrhotic patients ranged from 608 cells/*µ*L to 1024 cells/*µ*L, with a median of 784.5 cells/*µ*L. The CD4^+^ T-cell values in non-cirrhotic patients ranged from 688 to 1116 cells/*µ*L, with a median of 901.3 cells/*µ*L. The difference between the CD4^+^ T-cell values of the cirrhotic and non-cirrhotic patients was statistically significant, with a *p*-value of 0.003 (Figure 2a).

The CD8^+^ T-cell values in cirrhotic patients ranged from 508 cells/*µ*L to 1003 cells/*µ*L, with a median of 774.5 cells/*µ*L. The CD8^+^ T-cell values in non-cirrhotic patients ranged from 585 cells/*µ*L to 1013 cells/*µ*L, with a median of 786 cells/*µ*L. The difference between the CD8^+^ T-cell values of the cirrhotic and non-cirrhotic patients was not statistically significant, with a *p*-value of 0.417 (Figure 2b).

The CD4/CD8 ratio in cirrhotic patients ranged from 1.0 to 1.2, with a median of 1.0. The CD4/CD8 ratio in non-cirrhotic patients ranged from 1.0 to 1.4 with a median of 1.2. The difference between the CD4/CD8 ratios of the cirrhotic and non-cirrhotic patients was statistically significant, with a *p*-value of less than 0.001 (Figure 2c).

Within the group of non-cirrhotic and cirrhotic patients infected with HBV, there was a statistically significant direct correlation between CD95 and CD4^+^ T-cells (*r* = 0.042, *p* = 0.016), and between CD95 and the CD4/CD8 ratio (*r* = 0.123, *p* = 0.01). Within the group of cirrhotic patients infected with HBV, there was a statistically significant direct correlation between CD95 and fibrosis score (*r* = 0.021, *p* = 0.003). In this same group, there was a statistically significant inverse correlation between CD95 and CD8^+^ T-cells (*r* = −0.029, *p* = 0.016) (Table 2). We also found, within this same group, a statistically significant direct correlation between CD95L and the fibrosis score (*r* = 0.099, *p* = 0.003). There were statistically significant inverse correlations between the fibrosis score and the CD4^+^ T-cells (*r* = −0.280, *p* < 0.001), and between the fibrosis score and CD4/CD8 ratio (*r* = −0.455, *p* < 0.001) within the group of cirrhotic patients infected with HBV (Table 2).

**TABLE 2:** Correlation between CD95, CD95L, fibrosis score, CD4^+^ T-cells, CD8^+^ T-cells and CD4/CD8 ratio of hepatitis B virus-positive patients at Yaoundé General Hospital, Cameroon, September–December 2018.

Lymphocytic and hepatic parameters	CD95 (pg/mL)		CD95L (pg/mL)		Fibrosis score	
	*r*	*p*	*r*	*p*	*r*	*p*
CD4^+^ T-cells (cells/*µ*L)	0.042	0.016	−0.286	0.148	−0.280	0.009*
CD8^+^ T-cells (cells/*µ*L)	−0.029	0.016	−0.155	0.083	−0.039	0.426
CD4/CD8 ratio	0.123	0.01	−0.106	0.1	−0.455	< 0.001*
Fibrosis score	0.021	0.003	0.099	0.003	-	-

CD95L, CD95 ligand; *r*, Spearman’s correlation coefficient; *p, p*-value.

* , statistically significant > 0.05.

## Discussion

We sought to assess the implications of CD95-CD95L receptor-ligands in T-cell depletion and hepatic cytolysis, in patients with chronic HBV. We found that the plasma levels of CD95 and CD95L were significantly elevated in cirrhotic patients, compared to non-cirrhotic patients. The CD4/CD8 ratios were lower in cirrhotic patients, compared to non-cirrhotic patients. There were statistically significant correlations between the CD95 level and CD4^+^ T-cell count, between the CD95 level and the CD8^+^ T-cell count, between the CD95 level and CD4/CD8 ratio, between the CD95 level and fibrosis score, and between the CD95L level and fibrosis score.

The concentrations of CD95 and CD95L were higher in cirrhotic patients than in non-cirrhotic patients, with a statistically significant difference between the two groups. These results corroborate those of Peter et al.: they observed low constitutive expression of CD95 in non-cirrhotic patients with chronic HBV, compared to patients with cirrhosis related to HBV.^8^ The results obtained in this study agree with literature that CD95 is overexpressed during HBV infection.^9^ The expression of CD95 increases in response to a primary stimulus and this makes hepatocytes more susceptible to stimulation by CD95L. This hypothesis is supported by the observation that induction of CD95 expression occurs following chronic lymphatic histiocytic inflammation in different epithelial cells.^6^ These results suggest that liver damage in HBV-infected patients may primarily involve the destruction of hepatocytes by T-cells using the CD95-CD95L receptor-ligand system.

Immune system abnormalities are associated with T-cell depletion.^10^ We found statistically significant differences in CD4^+^ T-cell values between cirrhotic and non-cirrhotic patients. Mean values of CD4^+^ T-cells in cirrhotic patients were lower than those observed in non-cirrhotic patients. Also, the mean values of CD8^+^ T-cells in cirrhotic patients were higher than in non-cirrhotic patients. These abnormalities can be caused by abnormal regulation of the activation gene for the antigen 4 of cytotoxic T-cells, the lymphocytes 3, the immunoglobulin T domain and the mucin 3 domain, the cell death receptor 1, the CD244/2B4, the CD160 and by the T-cells immunoreceptor with immunoglobulin and immunoreceptor tyrosine-based inhibition motif domains.^11,12^

The average CD4/CD8 ratio in cirrhotic patients was similar to that found in 2016 by Yang et al.^13^ The CD4/CD8 mean ratio, however, was lower than that found in non-cirrhotic patients. This could reflect a possible increased immune depletion in cirrhotic patients, compared to non-cirrhotic patients.

We highlighted the existence of a statistically significant direct correlation between CD95 and CD4^+^ T-cells in HBV-infected patients, cirrhotic and non-cirrhotic. At the same time, there was a statistically significant, but inverse, correlation between CD95 and CD8. These results could be explained by the fact that the increase in activated CD8 T-cells is followed by their differentiation into cytotoxic T-cells. Cytotoxic T-cells are effector lymphocytes that cause the destruction of neighbouring cells having CD98 receptors on their surfaces, thus causing a decrease in the CD98 receptor. The statistically significant direct correlation between CD95 and the CD4/CD8 ratio observed in cirrhotic HBV-infected individuals could be due to the influence of the number of activated CD4^+^ T-cells carrying CD95 receptors, in the CD4/CD8 ratios. The increase in the number of activated CD4^+^ T-cells directly involves an increase in the production of CD95 receptors, which in turn increases the plasma concentration of CD95 receptors.

Although the correlation observed between CD95L and CD4^+^ T-cells and between CD8^+^ T-cells and the CD4/CD8 ratio was not statistically significant, both had inverse relationships in cirrhotic HBV-infected individuals. The CD95L level evolved in the opposite direction to the CD4^+^ T-cells, CD8^+^ T-cells and the CD4/CD8 ratio. This could be explained by the maintenance of the peripheral tolerance of T-cells activated by AICD mediated by the interaction between CD95 and CD95L.^6^ Indeed, over-activated effector T-cells are harmful to the immune system and must be removed from body tissues.

Also, there was a statistically significant direct correlation between the METAVIR fibrosis score and the CD95 and CD95L concentrations in these cirrhotic HBV-infected patients. This reflected an association between liver injury and activation of the CD95-CD95L apoptosis pathway. These results corroborate those obtained by Peter et al. in a study of CD95 receptor and ligand involvement in hepatic injury,^8^ where CD95 receptor expression was very high in hepatocytes in HBV-related cirrhosis.^12^ These results are also parallel to those obtained in the case of infections with HCV by Hayashi and Mita,^14^ where the expression of CD95 was upregulated according to the severity of inflammation of the liver.

There was a statistically significant inverse correlation between the fibrosis score and the CD4^+^ T-cell count as well as the CD4/CD8 ratio in cirrhotic HBV-infected patients. These results reflected an association between the specific cellular immune response via the activation of the CD4^+^ T-cells who are the ‘coordinators’ and the liver lesions that occurred; this therefore suggests that the destruction of hepatocytes in HBV infection would be induced by T-cells, using CD95-CD95L mediation.^12^ There was no statistically significant inverse correlation between the fibrosis score and CD8^+^ T-cell concentration in cirrhotic HBV-infected patients. These results show that expression levels of the constitutive receptor CD95 are functionally sufficient to mediate apoptosis of hepatocytes and T-cells.

### Limitations

Our study did not evaluate the activation of the CD95-CD95L signalling pathway during the evolution of HBV infection. We, therefore, propose a longitudinal study, which will evaluate the activation of the CD95-CD95L signalling pathway during the progression of the viral infection, from cultures of hepatocytes infected with HBV, and which will evaluate the inhibition of CD95 receptors as an immunotherapeutic method in patients with chronic HBV.

### Conclusion

The levels of CD95 and CD95L were higher in cirrhotic patients compared to non-cirrhotic patients. The CD4^+^ T-cell counts, CD8^+^ T-cell counts and CD4/CD8 ratios were respectively lower, higher, and lower in cirrhotic patients compared to non-cirrhotic patients. Thus, CD95-CD95L could be involved in T-cell depletion and hepatic cytolysis during the pathogenesis of chronic HBV and could potentially be used as biomarkers for immunological and hepatic monitoring in patients with chronic HBV.
